# APR-QKDN: A Quantum Key Distribution Network Routing Scheme Based on Application Priority Ranking

**DOI:** 10.3390/e24111519

**Published:** 2022-10-24

**Authors:** Liquan Chen, Ziyan Zhang, Mengnan Zhao, Kunliang Yu, Suhui Liu

**Affiliations:** 1 School of Cyber Science and Engineering, Southeast University, Nanjing 210096, China; 2 Purple Mountain Laboratories for Network and Communication Security, Nanjing 211118, China

**Keywords:** quantum key distribution, routing scheme, priority, trusted relay, QKD network

## Abstract

As the foundation of quantum secure communication, the quantum key distribution (QKD) network is impossible to construct by using the operation mechanism of traditional networks. In the meantime, most of the existing QKD network routing schemes do not fit some specific quantum key practicality scenarios. Aiming at the special scenario of high concurrency and large differences in application requirements, we propose a new quantum key distribution network routing scheme based on application priority ranking (APR-QKDN). Firstly, the proposed APR-QKDN scheme comprehensively uses the application’s priority, the total amount of key requirements, and the key update rate for prioritizing a large number of concurrent requests. The resource utilization and service efficiency of the network are improved by adjusting the processing order of requests. Secondly, the queuing strategy of the request comprehensively considers the current network resource situation. This means the same key request may adopt different evaluation strategies based on different network resource environments. Finally, the performance of the APR-QKDN routing scheme is compared with the existing schemes through simulation experiments. The results show that the success rate of application key requests of the APR-QKDN routing scheme is improved by at least 5% in the scenario of high concurrency.

## 1. Introduction

Quantum key distribution (QKD) technology [1], which encodes and transmits optical quantum signals, relies on the basic principles of quantum mechanics such as the Heisenberg uncertainty principle and quantum unclonable theorem to guarantee the unconditional security of secret key negotiation [2,3]. Once there is eavesdropping, both parties to the communication can detect it immediately. By connecting multiple point-to-point QKD systems to build a QKD network, users can be offered long-distance and networked key services [4,5,6]. With the continued evolution of quantum key networking technology, quantum keys have increasingly progressed closer to practicality. However, the current QKD networks are still in the experimental stage, and researchers have developed some simulation platforms [7], but their practical deployment is still difficult to realize due to complexity and high cost. To truly integrate into people’s daily lives, a variety of practical issues need to be taken into account. One important issue is that the dramatic increase in the number of network nodes as well as users will lead to increasingly complicated network topology [8], in which case an efficient routing scheme is highly critical.

QKD networks can be separated into three major categories: optical node based QKD networks, quantum relay-based QKD networks, and trusted relay-based QKD network [9,10,11]. Among these, trusted relay-based QKD networks that offer superior security and better scalability have been used in various actual QKD networks, and the feasibility of trusted relay technology in QKD networks has also been verified [12]. The routing scheme conducted in this paper uses trusted relay-based QKD networks as the underlying infrastructure. Compared to classical network routing, trusted relay-based QKD network routing has the following characteristics:

(1) Data processing: During packet forwarding, classical network routing only needs to read the address field of the packet and the locally stored routing table, whereas trusted relay-based QKD network routing also needs to encrypt and decrypt the information carried in the packets, which has a much higher data processing overhead.
(2) Forwarding capacity: The forwarding capacity of classical network routing depends mainly on the network bandwidth and is relatively fixed, while the forwarding capacity of trusted relay-based QKD network routing is also affected by the number of quantum keys stored inside the node and the associated links, so it is dynamically changing.
(3) Success rate: The success rate of classical network routing is mainly influenced by network congestion, while the success rate of trusted relay-based QKD network routing is influenced by both the classical channel bandwidth and the quantum channel bandwidth.

In addition, the link resources used by QKD networks in carrying user keys are considerably different from the bandwidth resources of classical networks. By combining the above descriptions, it can be concluded that the classical network routing scheme [13,14,15,16,17] cannot be directly applied to QKD networks. Therefore, a more efficient, flexible, and applicable routing scheme is needed.

There have been some studies on the routing problem in QKD networks, but most of them have not explored the relevant real-world scenarios, which are necessary for any application of quantum keys. Currently, most of the research results on the routing problem of trusted relay-based QKD networks have improved according to the characteristics of QKD networks by drawing on the proven classical network routing scheme. Yu et al. [18] transformed link resources in QKD networks into time-slice resources based on time-division multiplexing techniques, and the scheme developed a method to measure link time-slice continuity to reduce the secret key request conflicts caused by link resource fragmentation. However, the scheme does not start from a practical application scenario and ignores the difference in demand between applications and the particular case of high concurrency, which can lead the resource allocation to apply solely to the ideal case. Xu et al. [19] proposed a backtracking-based random routing scheme, which exploits backtracking points to prevent quantum key waste owing to repeated path selection. However, this scheme does not rely on the complete network topology when selecting paths but sacrifices the resource utilization of the network for path randomness. Additionally, the scheme ignores the problem of a considerable rise in the number of requests that occurs in practice, and the research is aimed at secret key requests of a single application, which would lead to a lack of practicality. 

Some routing schemes account for the changing demands of requests for secret keys and are better appropriate for practical applications. Cao et al. [20] first proposed the scheme to allocate keys according to security requirements in QKD systems, which sets up different security scenarios. However, their research mainly focuses on key allocation strategies for requests with different requirements, and lacks mechanisms to avoid request blocking in special scenarios. Ma et al. [21] proposed two RWTA schemes with flexible security level (FSL) and specific security level (SSL). The RWTA-FSL can make more link requests successfully established by lowering the security level, which has strong applicability. However, this scheme still has limitations in some special cases such as high concurrency scenarios. Chen et al. [22] proposed a QKD routing scheme based on application demand adaptation where different requirements of the application affect the designation of the routing scheme. Their scheme greatly improves the success rate of requests and provides high flexibility, but is not analyzed in special scenarios. Yu et al. [23] proposed a heuristic collaborative routing algorithm for partially-trusted relay QKD networks, which integrates consideration of relay and residual keys, but it lacks sufficient consideration of the differences among requests. In addition, some routing schemes [24,25,26,27] set up different request priorities for subsequent processing based on criteria such as security level, business differences, and request arrival time. These schemes satisfy the demand differences of requests in diverse areas and have relatively little limitation in terms of efficiency improvement consideration.

The above studies lack consideration for highly concurrent scenarios. In this respect, some routing schemes use dynamic information and are more adapted to rapidly changing QKD network environments, thereby complementing this deficiency. Yang et al. [28] propose a dynamic routing scheme with a link state update mechanism, but key consumption is performed locally. Yang et al. [29] proposed a key-aware routing scheme based on the ability to predict the number of remaining keys in a link. Yao et al. [30] modified and optimized the OLSR protocol, designed a key recovery capability metric, and then proposed a more efficient QKD routing scheme with a link state awareness mechanism. However, these aforementioned schemes have room for optimization in the capacity to fulfill the needs of different request demands.

In summary, the following problems remain in the present study on QKD network routing algorithms. Firstly, the secret key requests of all applications are treated equally, without considering the difference in the importance of different requests throughout the actual application. This can lead to a backlog of delayed requests with high-security requirements in high concurrency circumstances, thus causing a worsening of the overall network quality of service. Secondly, when a large number of secret key requests occur in a short period, requests with extremely high secret key demand may be prioritized based on the traditional first-come first-serve (FCFS) algorithm request processing strategy. This would result in a deviation in network resource allocation and a reduction in the request success rate of the overall network. Lastly, when the network resources are insufficient, the existing schemes adopt two strategies: direct rejection or never rejection. Direct rejection may lead to frequent repeated secret key requests. Never rejection may cause the application to block due to waiting for the secret key response, and the progress of secret key request processing will not be known for a long time.

To tackle the aforesaid problems, this paper proposes a QKD network routing scheme based on the application priority ranking (APR-QKDN). Contributions are summarized as follows:

1. Considering the different importance and communication urgency of various applications in the actual use, we set a priority judgment criterion for application key requests in the scheme to quantify the priority of requests being processed.
2. For specific scenarios of high concurrency, a fixed amount of secret key requests of applications reached in a short period are ranked by priority rather than merely relying on the order in which the key requests are reached. The requests will be processed later according to the ranking result.
3. Depending on the permissible delay range for applications, secret key requests of applications with route failure are given a delayed retry instead of simply outright rejection and excessive infinite waiting.

Through simulation, it is proven that our scheme can acquire a greater request success rate compared with other schemes, better utilize the limited resources in the QKD network, and improve the actual quality of service of the quantum network. Accordingly, our work serves to advance the practical implementation of QKD technology.

The remainder of this paper is organized as follows: In Section 2, the system model and the related definitions are presented. In Section 3, we describe the proposed APR-QKDN scheme in detail, along with the calculation method of the priority judgment criterion. The performance outcomes compared with other schemes in diverse aspects are evaluated in Section 4. In Section 5, we summarize the paper.

## 2. QKD Network Model

### 2.1. System Model

**Figure 1:**
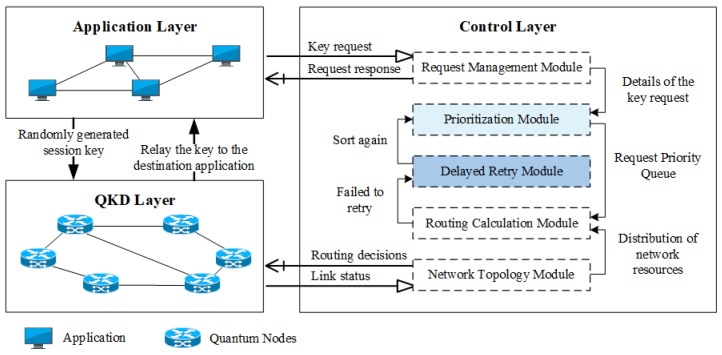
QKD network system model of APR-QKDN.

The trusted relay-based QKD network system used in this paper contains three layers: the application layer, the controller layer, and the QKD layer. The system model is illustrated in Figure 1.

1. Application layer: The application layer consists of various application entities involved in data transfer. This layer is the service body of the QKD network as well as the bridge between the QKD network and real users. The application in this layer will initiate a secret key request at any moment. This secret key request is submitted to the controller, and when the controller gets the secret key request, the application enters the blocking and waiting phase. Only when the application receives the request response information back from the controller can it begin the subsequent key generation phase.
2. Control layer: The control layer contains five modules: request management module, priority ranking module, delay retry module, network topology module, and route calculation module. The request management module is used to receive the secret key request of applications and send the details contained in requests to the priority ranking module. Subsequently, the priority ranking module will rank the huge number of requests arriving within a short period by the present resource status of the QKD network and the priority judging criterion defined in advance. After finishing the ranking, the priority ranking module delivers the request priority queue to the route calculation module for path selection, which is carried out under a specified strategy. When a request meets with route failure owing to insufficient resources, the request is delivered by the route calculation module to the delay retry module where the application will determine whether to rejoin the request queue according to its acceptable delay range. If the current delay is not exceeded, the request can be handled again by the priority module until the route is successful or timeout. The routing calculation module interfaces with the network topology module to gather the network resource status and give a full network topology for path selection.
3. QKD layer: The quantum nodes in the QKD layer report the resource storage and operation status to the controller’s network topology module in real-time. When the controller’s route calculation module completes the path selection, it distributes the corresponding routing decision to each quantum node in the QKD layer quantum node. Quantum nodes would update the local routing table entries. When the application receives the key request response, it transmits the session key relay to the target application through each quantum node in the QKD layer. Finally, the two communicating parties would have the same session key.

### 2.2. Relevant Definitions of the Model

The APR-QKDN routing scheme serves specific scenarios with considerable variances in application secret key demands and establishes the priority judgment criterion for different requests. Therefore, the QKD network model used in this paper defines the application priority metrics. Meanwhile, the total key demand and the key update rate of the QKD network are evaluated. The nomenclature included in the model is defined as described below.

1. Quantum link: a virtual link between neighboring quantum nodes abstracted for QKD, the underlying physical form is the combination of quantum channel and measurement-based channel, the process of QKD contains the information transmission of these two channels.
2. Link time slice: each quantum link has a particular key generation rate that can be divided, meaning that the key generation cycle can be divided evenly to produce a time slice. Later, for different secret key requests of applications, the time slice is allocated on demand, i.e., occupying a period in the key generation cycle for updating its session key.
3. Link key: the secret key generated by each quantum link is called the link key. It is generated by the negotiation of neighboring quantum nodes.
4. Link key pool: the link key pool is a virtual concept that manifests as a pair of local key pools in neighboring quantum nodes. The quantum keys generated by the idle link time slice are stored in the link key pool, which can directly provide key services for applications and support the case of insufficient link time slice.
5. Application priority: depending on the degree of importance, each application will have a distinct priority. The same application may have different levels of data transmission urgency at different times, thereby the application priority might change dynamically. A higher priority secret key request of an application signifies that the application has a higher security requirement or the application urgently needs to obtain a key for confidential communication.
6. Total key request: the meaning of total key request is the total amount of key requests of an application. It plays a significant part in determining the request priority. A large total key request of an application suggests that there would be more difficulties to allocate resources for the current application and comprehensive consideration of the existing network resource state is needed.
7. Key update rate: it is similar to the total key request but influences more the consumption of time slices of the quantum link rather than the consumption of the number of keys remaining in each quantum node. The higher key update rate that an application demand indicates, the more time slice resources that the application needs to occupy. Nevertheless, the key generation rate of the link is limited. Once the time slice resources are occupied by an application, they are difficult to be released in a short time.

## 3. APR-QKDN Routing Scheme

To address the problems of existing QKD network routing schemes, we propose a QKD network routing scheme based on application priority ranking (APR-QKDN) in this paper. Considering the situation that a large number of requests arrive in a short period, APR-QKDN sets a reasonable priority judgment criterion of requests and adjusts the processing order of requests so that the routing scheme can satisfy as many application requests as possible with the limited resources of the QKD network. Moreover, APR-QKDN grants a delayed retry chance to the request with route failure instead of rejecting it straightaway, intending to improve the overall service quality of the routing scheme. Since we sort requests based on priority, our solution is more suitable for scenarios with high concurrency and large gaps in application requirements than previous solutions. Since the range of sorting can be adjusted and the priority criteria can be customized and adjusted according to the usage requirements, our solution has high flexibility and low overhead, such as the ability to fall back to FCFS for non-high concurrency scenarios.

In this section, the priority judgment criterion of requests and routing scheme will be explained in detail. Table 1 shows the symbols to be used and their meanings. Among them, the application priority is not established by the application itself, it is appraised and defined by the route based on the information carried by the request. Note that the application priority ($R_{\mathrm{pr}\_\mathrm{self}}$) is different from the priority judgment criterion ($R_{\mathrm{pr}}$): the application priority is one of the influencing variables of the priority judgment criterion, while the priority judgment criterion determines the order of requests.

### 3.1. Priority Judgment Criterion of Requests

In practical usage circumstances, both the importance of secret key requests and the specific secret key demands are diverse in different applications; performing the FCFS strategy cannot deliver adequate service quality and high network resource utilization. Under the foregoing scenario, APR-QKDN sets a reasonable priority judgment criterion based on the application priority and secret key demands, then decides the order of subsequent key requests according to this criterion.

The priority judgment criterion considers three factors: the application priority, the total key request of applications, and the key update rate of applications. How to weigh the importance of these factors is a crucial issue. The specific evaluation formula is as follows:

(1) $$R_{\mathrm{pr}}{\;=\;\alpha \;\times \;R}_{\mathrm{pr}\_\mathrm{self}}{\;+\;\beta \;\times \;\mathrm{Key}}_{\mathrm{vol}}{\;+\;\gamma \;\times \;\mathrm{Key}}_{\mathrm{UR}}$$

In Equation (1), $R_{\mathrm{pr}}$ denotes the priority indicator of the request, and the larger the value of $R_{\mathrm{pr}}$, the lower the priority; $R_{\mathrm{pr}\_\mathrm{self}}$ is set to the value range of [1, 5]; ${\mathrm{Key}}_{\mathrm{vol}}$ donates the total key request; ${\mathrm{Key}}_{\mathrm{UR}}$ represents demands of key update rate. For each factor, a larger value implies a lower application priority and a higher resource requirement, and the requests are processed in a comparatively lower order as a result. *α*, *β*, *γ* signify the weights of the three factors, respectively, and the larger the weight, the higher the impact on the priority of the request. The configuration of the weights needs to be altered dynamically according to the demands of applications, and the following are specific application cases:

1. The total key request is less than the minimum number of remaining link keys in the link set: the request can be satisfied by the remaining link key solely and does not have to be allocated link time slices. Therefore, the priority judgment criterion of the request does not need to consider the effect of the key update rate. In this case, *γ* should be set to 0, and the priority judgment criterion can be expressed as follows:
  (2) $$R_{\mathrm{pr}}{\;=\;\alpha \;\times \;R}_{\mathrm{pr}\_\mathrm{self}}{\;+\;\beta \;\times \;\mathrm{Key}}_{\mathrm{vol}}$$
2. The total key request is higher than the maximum number of remaining link keys in the link set: the request can only obtain a key by being allocated a link time slice. Therefore, the priority judgment criterion of the request does not need to consider the effect of the total key request. In this case, *β* should be set to 0, and the priority judgment criterion can be expressed as follows:
  (3) $$R_{\mathrm{pr}}{\;=\;\alpha \;\times \;R}_{\mathrm{pr}\_\mathrm{self}}{\;+\;\gamma \;\times \;\mathrm{Key}}_{\mathrm{UR}}$$
3. The total key request is between the minimum and the maximum number of remaining link keys in the link set: the specific approach to satisfy the application request cannot be determined directly. It is necessary to consider the total key request and the key update rate. In this case, ${\mathrm{Key}}_{\mathrm{vol}}$ and ${\mathrm{Key}}_{\mathrm{UR}}$ is of the same importance which can be derived as *β* = *γ*, and the priority judgment criterion can be expressed as Equation (1).

In summary, the priority judgment criterion is influenced by three factors, and the weights of the contributing factors fluctuate as the application’s features and requirements change.

### 3.2. Routing Scheme

APR-QKDN decides the routing order of requests according to the priority judgment criterion. Since there is a time interval between requests, it is necessary to set a suitable number of requests processed at one time ($R_{\mathrm{pr}\_\mathrm{num}}$), and prioritize the $R_{\mathrm{pr}\_\mathrm{num}}$ requests accumulated in a short period each time. $R_{\mathrm{pr}\_\mathrm{num}}$ should not be set too large, thereby preventing the accumulation of too many requests leading to individual timeout failures. It should not be set too small, either; otherwise it will cause the routing algorithm to degenerate into the standard FCFS strategy. When the prioritization of requests is done, the requests are routed progressively, as described in the following phases. We use the route management center to represent the controller node at the control layer.

**Step 1** The application sends the secret key request ${\mathrm{Key}}_{\mathrm{req}}$ to the route management center, including the source node ${\mathrm{Node}}_{S}$, the destination node ${\mathrm{Node}}_{D}$, total key request ${\mathrm{Key}}_{\mathrm{vol}}$, key update rate ${\mathrm{Key}}_{\mathrm{UR}}$, maximum acceptable delay for requests $R_{\mathrm{delay}}$, and the maximum delay allowed by the system $R_{\max\_\mathrm{delay}}$.

**Step 2** The routing management center calculates the priority judgment criterion of each received request and adds the requests whose maximum acceptable delay is within the allowable range of the system (i.e., $R_{\mathrm{delay}}\leq R_{\max\_\mathrm{delay}}$) to the request priority queue ${\mathrm{List}}_{\mathrm{req}}$. The order of requests recorded in ${\mathrm{List}}_{\mathrm{req}}$ satisfies the law of decreasing priority. Each time a request is added to the queue, the number of requests ($R_{\mathrm{cur}\_\mathrm{num}}$) accumulated in the current queue is incremented.

**Step 3** When the number of requests in ${\mathrm{List}}_{\mathrm{req}}$ reaches the predefined $R_{\mathrm{pr}\_\mathrm{num}}$ (i.e., $R_{\mathrm{cur}\_\mathrm{num}}{=R}_{\mathrm{pr}\_\mathrm{num}}$), the routing management center processes the requests in ${\mathrm{List}}_{\mathrm{req}}$ in sequence, and the processing order is the priority order of the requests. 

**Step 4** Filter the links according to the total key request of applications, and initialize *Flag* to 0. Iterate over the set of links; if the remaining key amount of a link can fulfill the application demands (i.e., ${\mathrm{Link}}_{\mathrm{RKV}}\;\geq {\;\mathrm{Key}}_{\mathrm{vol}}$), then add it to the list of alternate links (${\mathrm{List}}_{\mathrm{link}}$).

**Step 5** According to the source node ${\mathrm{Node}}_{S}$ and the destination node ${\mathrm{Node}}_{D}$ of secret key requests, viable paths are searched in the list of alternate links. Once a feasible path is found, it will be added to the list of optional paths; in that case, skip to Step 6. If there is no optional path, the status is further determined by judging the *Flag*: if the *Flag* is 0, it means that the number of remaining link keys cannot meet the secret key demand of the application, and it is necessary to route according to the available time slice of the link; in that case, skip to Step 7. If *Flag* is 1, the number of remaining link keys and the available link key generation rate cannot supply the secret key requirement of the application, which means the route failure; in that case, skip to Step 8. 

**Step 6** The routing management center calculates the total link cost of all feasible paths and selects the path with the lowest overall link cost as the optimal path. Subsequently, the associated routing table entries (a sequence of quantum nodes starting with ${\mathrm{Node}}_{S}$ and ending with ${\mathrm{Node}}_{D}$) are generated according to the selected optimal path and distributed to each quantum node in the optimal path. After that, these quantum nodes update their routing forwarding tables which are needed to determine the next-hop nodes for secret key relaying.

**Step 7** Filter the links according to the key update rate of applications and set *Flag* to 1. Iterate over the set of links; if the available link key generation rate (available time slice) can fulfill the application demands (i.e., ${\mathrm{Link}}_{\mathrm{AKGR}}\;\geq {\;\mathrm{Key}}_{\mathrm{UR}}$), then add it to the list of alternate link (${\mathrm{List}}_{\mathrm{link}}$), afterward; skip to Step 5.

**Step 8** Evaluate whether a request with route failure is eligible for a delayed retry. If the number of request retries is within 5 and the current delay of request does not exceed the maximum acceptable delay for requests (i.e., $N_{\mathrm{retry}}\leq 5$ and $R_{\mathrm{cur}\_\mathrm{delay}}{<R}_{\mathrm{delay}}$), the request would be re-added to the request priority queue, and the number of request retries is increased. Otherwise, the response of route failure would be returned to the application. 

**Figure 2:**
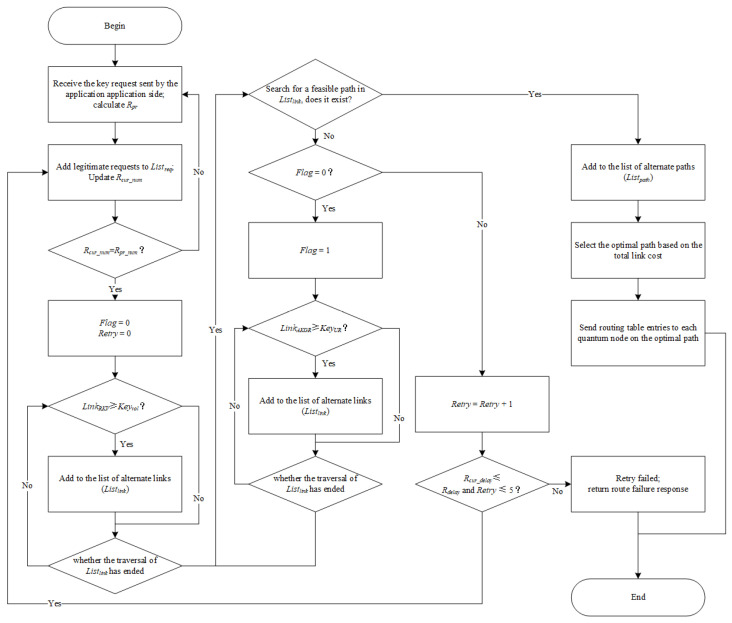
Algorithm flow chart.

According to our experiment, we set the number of request retries at 5 in Step 8. It can be modified to satisfy the actual situation. Figure 2 illustrates the flow of the program.

## 4. Simulation Experiment and Analysis

**Figure 3:**
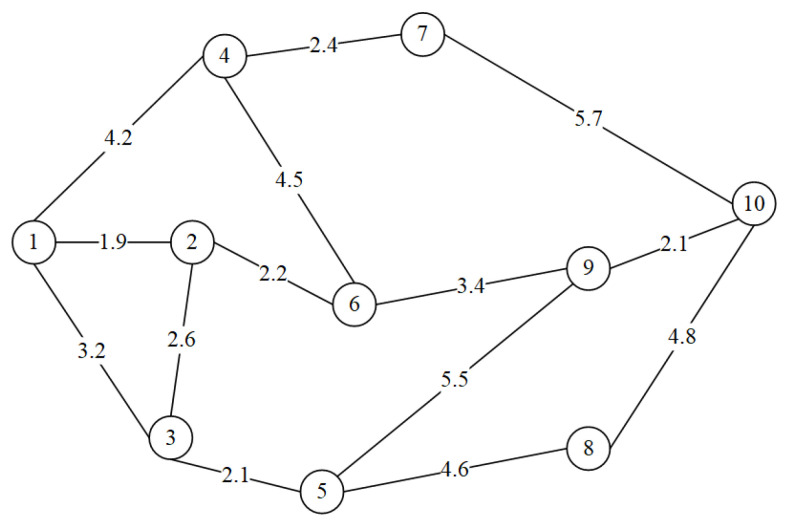
Network simulation topology diagram. (Numbers 1–10 represent different network nodes).

To evaluate the performance of the APR-QKDN routing scheme proposed in this paper, we complete the performance comparison between different schemes through simulation experiments. The network topology used for the experiments is displayed in Figure 3. It is comprised of 10 nodes and 14 links. The lengths of its links are labeled in the figure. The links are measured in kilometers.

The comparison schemes employed in the simulation experiments are the KoD [20], the RWTA-FSL [21], and the ADA-QKDN [22] scheme, all of which do not consider the likelihood of enormous differences between application secret key requests of applications and hence adopt the typical FCFS technique. Faced with the highly concurrent scenario of a large number of key requests arriving in a short period, the above three schemes, although simpler in deciding the order of request processing, will suffer from certain performance drawbacks and make it difficult to utilize the limited resources to improve the overall request success rate.

Our APR-QKDN routing scheme targets the specific scenario with high concurrency and substantial differences in secret key demand between applications, hence it is necessary for this particular scene to be reproduced when comparing the performance of each scheme. In each simulation, the secret key requests of applications vary considerably. Consequently, there is no means to use the total key request and key update rate, which reflect the demand of applications, as fixed experimental parameters. Only application-independent environmental parameters can be changed dynamically. 

The experimental parameters considered for this simulation are the key pool expansion multiplier, interval of requests, and link key generation rate. The success rate of requests, which represents the request processing capability of the scheme, is the most essential service quality evaluation metric. The key efficiency is the ratio of the total key demand of a request to the total number of keys consumed to complete that request. A larger key efficiency rate indicates that the request itself has a larger share of the key demand and a lower level of quantum key waste. In a single experiment, one of the experimental parameters will be altered dynamically, and the success rate of requests under that particular parameter will be utilized as the performance assessment index of the four schemes. In the following different circumstances, the three parameters are used as a variable in turn. 

### 4.1. Performance Comparison under Different Key Pool Expansion Multiplier

In this circumstance, the key pool expansion multiplier varies in the range of [50, 1000], the key update rate varies in the range of [128, 1,280,000] bps, the Poisson distribution parameter of the interval of requests is 100 ms, the number of requests processed at one time ($R_{\mathrm{pr}\_\mathrm{num}}$) is 5, weights *α*, *β* and *γ* that influence total key request are all set to 0.33, the application duration satisfies a uniform distribution in the range of [10, 600] s, the initial capacity of the key pool is 12,800 bit.

**Figure 4:**
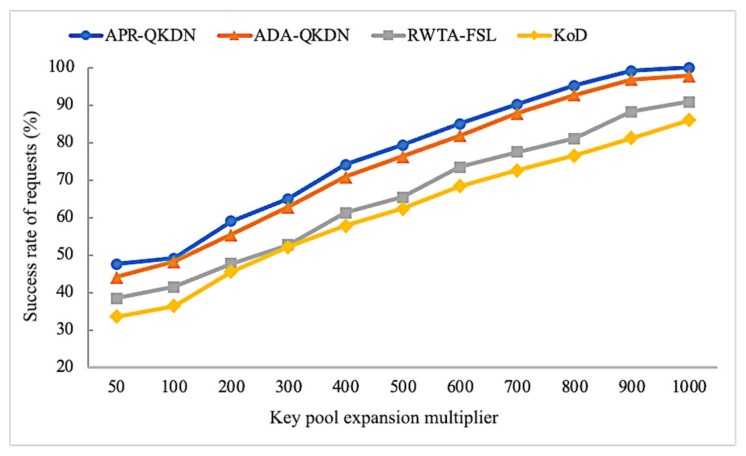
Impact of key pool expansion multiplier on the success rate of requests for each scheme.

As can be seen in Figure 4, the success rate of requests of APR-QKDN and the other three schemes show an increasing trend as the key pool expansion multiplier increases. The reason is the number of keys that can be stored locally by the quantum nodes will increase as the key pool expansion multiplier increases, making the network resources gradually abundant and thus able to satisfy more requests. Simultaneously, the success rate of requests for the APR-QKDN scheme has a significant performance improvement compared with KoD and RWTA-FSL for a certain key pool expansion multiplier. The difference between APR-QKDN and KoD is maintained at around 15%, and the difference with RWTA-FSL is about 10%, which is due to the appropriate path costing strategy chosen by APR-QKDN. Our scheme achieves a performance gain of roughly 5% in the success rate of requests compared to ADA-QKDN, which is attributed to the adaptive prioritization of application requests. 

**Figure 5:**
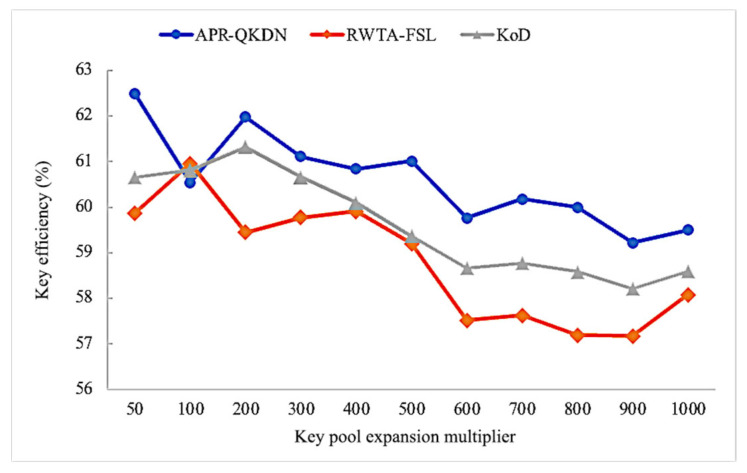
Impact of key pool expansion multiplier on key efficiency for each scheme.

As shown in Figure 5, with the gradual increase of key pool expansion multiplier, the overall key efficiency rate of each scheme displays a falling trend. When the key pool expansion multiplier is large, the key pool capacity of the node will be much larger than the total amount of keys requested by the application, each scheme will give priority to allocate the remaining local key amount and may overlook the paths with sufficient link time slice resources but lesser hop count. Therefore, it is vital to set the proper key pool capacity and not to pursue the key pool expansion. Although KoD and RWTA-FSL both execute routing based on the shortest path algorithm, the key efficiency of APS-QKDN still has some advantages, but the overall difference is not significant. Among them, our scheme maintains a gap of roughly 3% with RWTA-FSL and has about 2% improvement compared to KoD.

### 4.2. Performance Comparison under Different Intervals of Requests

In this circumstance, the interval of requests varies in the range of [1, 2000] ms, the key update rate varies in the range of [128, 1,280,000] bps, the key pool expansion multiplier of KoD is [1, 50, 100], the number of requests processed at one time ($R_{\mathrm{pr}\_\mathrm{num}}$) is 5, weights *α*, *β* and *γ* that influence total key request are all 0.33, the application duration satisfies a uniform distribution in the range of [10, 600] s, the initial capacity of the key pool is 12,800 bit.

**Figure 6:**
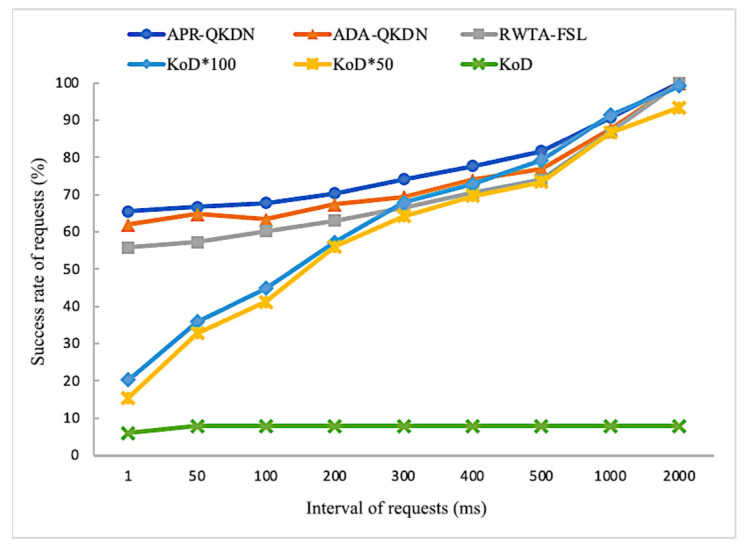
Impact of the interval of requests on the success rate of requests for each scheme.

As shown in Figure 6, the success rates of requests of APR-QKDN, ADA-QKDN, RWTA-FSL, and unscaled KoD change slowly with an increasing interval of requests, and APR-QKDN has a persistent performance advantage over other schemes when the interval of requests is small. Compared with ADA-QKDN, our scheme has about 5% improvement in the success rate of requests; compared with RWTA-FSL, it has about 10% improvement; compared with unexpanded KoD solution, it has at least 60% improvement; compared with KoD solution with 100 times expansion, it has about 30% improvement on average when the request interval is small. When the request interval exceeds 1000 ms, the performance of the highly expanded KoD scheme gradually surpasses that of APR-QKDN, mainly due to the key replenishment under the long idle period and the solid equipment foundation built by the large-capacity key pool. However, it would also lead to more pressure on the construction of network facilities. 

**Figure 7:**
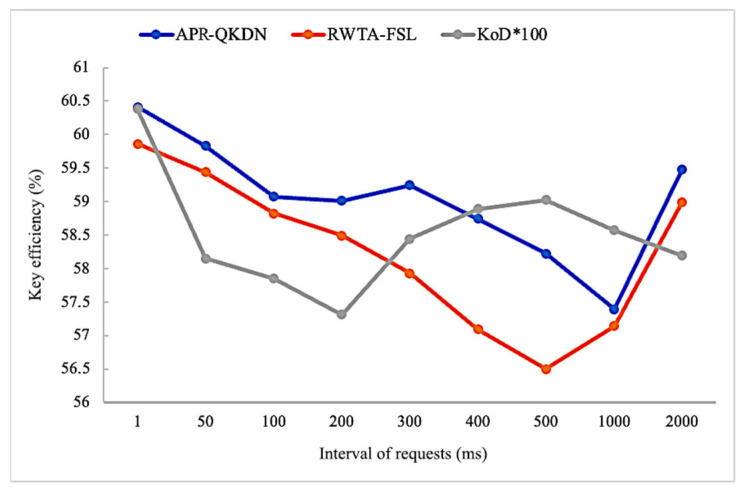
Impact of the interval of requests on key efficiency for each scheme.

As shown in Figure 7, during the dynamic change of the request interval, the key efficiency of each scheme shows fluctuation without a clear trend of change. This is because the change of request interval mainly changes the remaining key amount in the key pool while the key pool capacity has a certain gap compared with the total key demand of the application, and the key replenishment within the request interval has no way to meet a large number of requests, so the impact on the number of path hops is not significant and does not affect the key efficiency considerably. From the perspective of average key efficiency, APS-QKDN has at least 2% performance improvement compared with RWTA-FSL and KoD scheme with 100 times expansion.

### 4.3. Performance Comparison under Different Link Key Generation Rate

In this circumstance, the link key generation rate varies in the range of [1, 150] kbps, the key update rate varies in the range of [128, 1,280,000] bps, the Poisson distribution parameter of the interval of requests is 100 ms, the key pool expansion multiplier of KoD is [1, 50, 100], the number of requests processed at one time ($R_{\mathrm{pr}\_\mathrm{num}}$) is 5, weights *α*, *β* and *γ* that influence total key request are all 0.33, the application duration satisfies a uniform distribution in the range of [10, 600] s, the initial capacity of the key pool is 12,800 bit.

**Figure 8:**
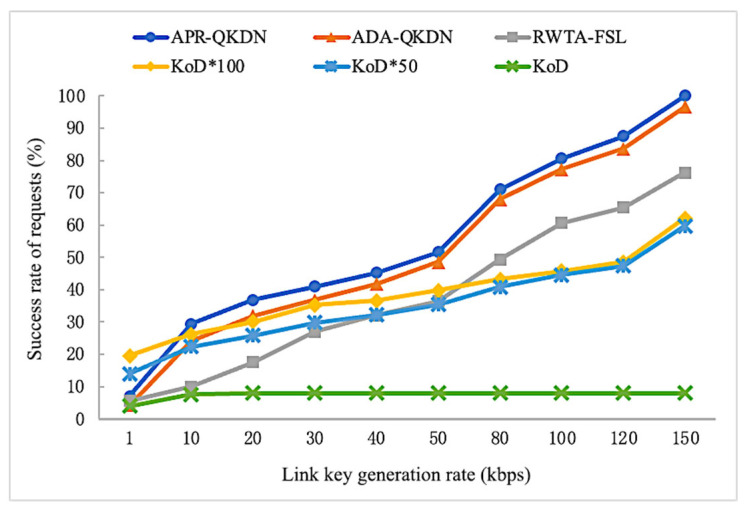
Impact of link key generation rate on the success rate of requests for each scheme.

As shown in Figure 8, the success rates of requests corresponding to APR-QKDN, ADA-QKDN, and RWTA-FSL increase significantly with the growth of the link key generation rate, and the changing trends of the three are similar. In the case of KoD without expansion, the success rate of requests remains stable and does not have an increasing tendency. The success rate of requests corresponding to the highly expanded KoD scheme progressively climbs with the growth of the link key generation rate, but the changing trend is relatively moderate. Our scheme offers various advantages over the other three schemes in terms of the success rate of requests, which can improve by around 5% compared to ADA-QKDN and about 20% compared to RWTA-FSL. The APR-QKDN is around 30% better than the KoD scheme with 100x extension; the difference is even greater compared to the unexpanded form of KoD. 

**Figure 9:**
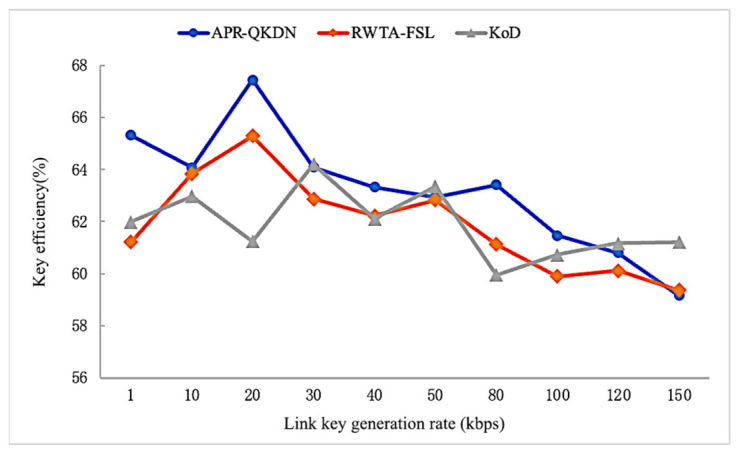
Impact of link key generation rate on the success rate of requests for each scheme.

As shown in Figure 9, the key efficiency of our scheme as well as RWTA-FSL shows a fluctuating downward trend as the link key generation rate grows. To analyze the reason, the growth of the link key generation rate leads to a reduction of link time slices required for a single request, each link can serve more key requests in one-time cycle, and the resources of some key nodes may be allocated to the earlier requests, so the feasible paths of subsequent requests are more likely to be bypassed. Since the growth of the link key generation rate in the KoD scheme has an impact on the key pool replenishment limited by the short request interval, and the impact on the network resources can be ignored, the key efficiency rate of this scheme has no obvious trend and shows a random fluctuation overall. The average key efficiency of the APS-QKDN scheme is 63% during the change of link key generation rate, which is at least 2% higher than that of RWTA-FSL and KoD.

In general, the APR-QKDN routing scheme maintains good performance during the dynamic changes of the three experimental parameters with some performance improvement compared to the other three schemes and is more suitable for specific scenarios with high concurrency and large inter-application demand differences. From the above experimental results, we find that the request success rate of APR-QKDN has more opportunities to improve when the link key generation rate keeps increasing. The link key generation rate has a good prospect, so it is feasible to improve the request success rate of this scheme by increasing the link key generation rate.

In addition, since different requests have different application priorities, how to integrate the importance of requests to evaluate the service quality of the scheme comprehensively and design new additional evaluation metrics is a question to be considered in our future work.

## 5. Conclusions

In this paper, we focus on the research of QKD network routing schemes in specific scenarios with considerable disparities in demand between applications and high concurrency, and discuss the system model and application characteristics of the proposed APR-QKDN scheme. The priority judgment criteria and the delayed retry mechanism adopted by the APR-QKDN scheme are explained in detail. On the one hand, the application priority is adaptively and dynamically updated according to the characteristics of applications and the current network environment, enabling the system to adjust a suitable processing order for an immense number of requests arriving in a short period. On the other hand, considering that the application itself has a certain delay tolerance and there are unreasonable request rejection strategies in existing routing schemes, our scheme adds a delayed retry mechanism, which not only enables the application to follow up on the request processing progress in time but also avoids frequent and repeated network data transmission. Finally, the performance of the APR-QKDN scheme is experimentally compared with KoD, RWTA-FSL, and ADA-QKDN schemes through simulation. The experimental results demonstrate that the APR-QKDN scheme has a certain performance improvement compared to the other three schemes. The success rate performance improvement is the most noticeable compared to KoD, reaching up to 60%; compared to RWTA-FSL, it can enhance around 15%, and the performance gap between APR-QKDN and ADA-QKDN is about 5%. It is shown that the proposed APR-QKDN scheme has good performance in specific scenarios with intensive demands and big variances between requests, which is important for increasing the service quality of QKD networks. 

## Figures and Tables

**Table 1:** Symbols and their meanings.

Symbols	Meanings
$R_{\mathrm{pr}}$	priority judgment criterion of requests
$R_{\mathrm{pr}\_\mathrm{self}}$	application priority
${\mathrm{Key}}_{\mathrm{vol}}$	total key request
${\mathrm{Key}}_{\mathrm{UR}}$	key update rate
$N_{\mathrm{retry}}$	number of request retries
$R_{\mathrm{delay}}$	maximum acceptable delay for requests
$R_{\max\_\mathrm{delay}}$	maximum delay allowed by the system
$R_{\mathrm{cur}\_\mathrm{delay}}$	current delay of the request
$R_{\mathrm{pr}\_\mathrm{num}}$	number of requests processed at one time
$R_{\mathrm{cur}\_\mathrm{num}}$	current number of accumulated requests
${\mathrm{Key}}_{\mathrm{req}}$	secret key request of applications
${\mathrm{Node}}_{S}$	source node of secret key requests
${\mathrm{Node}}_{D}$	destination node of secret key requests
${\mathrm{List}}_{\mathrm{req}}$	request priority queue
${\mathrm{List}}_{\mathrm{link}}$	list of alternate links
${\mathrm{List}}_{\mathrm{path}}$	list of optional paths
${\mathrm{Link}}_{\mathrm{RKV}}$	number of remaining link keys
${\mathrm{Link}}_{\mathrm{AKGR}}$	available link key generation rate

## Data Availability

Not applicable.
